# Using the internet to obtain dermatological information on patients from the public health network: a cross-sectional study^⋆^

**DOI:** 10.1016/j.abd.2020.12.015

**Published:** 2022-06-04

**Authors:** Bianca Latance da Cruz, Arthur Cesar dos Santos Minato, Ioana Bittencourt Mourão, Dayane Neres Pereira, Miguel Huckembeck de Oliveira, Juliano Vilaverde Schmitt

**Affiliations:** Department of Infectology, Dermatology, Diagnostic Imaging and Radiotherapy, Faculty of Medicine, Universidade Estadual Paulista, Botucatu, SP, Brazil

Dear Editor,

The internet is a powerful and accessible media, it can also offer knowledge in the health area so that users may understand the possible involvement by a disease and its outcomes.^1^ Previous studies have shown that women, young people, university students, and individuals with higher incomes are more likely to seek health information on the internet.^2^ However, there have been few studies on the influence of research on dermatological diseases. Thus, given the Brazilian profile of intensive use of the internet, including for health information, it is necessary to study the way people use this tool in their daily lives.^1^, ^3^

Considering the abovementioned facts, this study aimed to evaluate the prevalence of internet access to obtain information on skin health among dermatological patients, their demographic and search profile, and their associations with internet use, as well as the interactions of the results with dermatological care.

This is a cross-sectional, descriptive and exploratory study, carried out with patients from a public hospital in the hinterland the state of São Paulo, interviewed between July and September 2019. Participants were recruited for convenience in the waiting areas for scheduled outpatient dermatological care.

Patients over 18 years of age who were literate and had no communication problems, cognitive impairment, or psychiatric illness, that prevented the interview from being conducted, were included.

Data collection was carried out using a two-part investigation protocol: the first on patient demographics and the second on the use of the internet in health-related searches.

The study was approved by the institutional Ethics Committee (Counsel n. 3,661,913).

Continuous variables were analyzed as bivariate data using the parametric Student's *t* test after normal distributions were assessed using the Shapiro-Wilk test. Categorical variables were compared using the chi-square or Fisher's exact tests according to the lowest number of events in each analysis.

The variables indicating the type of researched information and the type of tool used in the search were analyzed using hierarchical clustering, linkages between groups, and Euclidean distance, represented by dendrograms using centroid linkage.

The minimum sample size consisted of 130 individuals for an exploratory analysis with up to 12 variables.

The categorical data were represented as absolute numbers and/or percentages, and non-categorical data as means and standard deviations.

The association between searching for skin-related health information on the internet and the other demographic variables was evaluated in a bivariate manner, and, subsequently, the significant variables were included in a multivariate logistic regression model.

Two-tailed values ​​of p ≤ 0.05 were considered significant.

Table 1 describes the socioeconomic data and the internet use of the 148 patients who agreed to participate in the research protocol.

**Table 1:** Demographic and socioeconomic data of dermatological patients participating in the survey on the use of the internet to obtain information on skin health.

Variable	n (%)
Age (mean and SD)	44.03 (15.26)
Sex	
Female	105 (70.9)
Male	43 (29.1)
Level of Schooling	
Incomplete Elementary School	28 (18.9)
Complete Elementary School	20 (13.5)
Complete or incomplete High School	57 (38.5)
Complete or incomplete Higher Education	43 (29.1)
Income	
Up to 1,000 reais	50 (33.8)
1,000 to 3,000 reais	74 (50)
More than 3,000 reais	24 (16.2)
Time commuting	
< 20 minutes	38 (25.7)
20 to 60 minutes	44 (29.7)
> 60 minutes	66 (44.6)
Access to the internet at home	131 (88.5)
Possession of a smartphone	135 (91.2)
Use for health-related issues	113 (76.4)
Use for skin health-related issues	102 (68.9)
Where do you seek information?	
Social networks	26 (17.6)
Message exchange	4 (2.7)
Search engines	81 (54.7)
Blogs	17 (11.5)
News sites	2 (1.4)
Government sites	3 (2)
Moment in relation to consultation	
Before	49 (60.8)
After	27 (45.9)
Discussion with the doctor about the information from the internet	
Never / rarely	75 (50.7)
Sometimes	23 (15.5)
Frequently	13 (8.8)
Almost always	8 (5.4)
Reason	
Diagnosis	42 (28.4)
Alternative treatment	40 (27)
Side effects	25 (16.9)
Prevention	17 (11.5)
Prognosis	24 (16.2)
Others	12 (8.1)
Device	
Smartphone	101 (68.2)
Desktop	17 (11.5)
Tablet and others	3 (2)
Conflicts with medical treatment	34 (23)
Influence on treatment	28 (18.9)
Provoked alteration	4 (2.7)
Confidence	
Very little	25 (16.9)
Little	36 (24.3)
Medium	41 (27.7)
Very much/ total	6 (4.1)
Depends on the source	9 (6.1)

Table 2 depicts the association of demographic variables with having obtained dermatological health information from the internet. Obtaining this information was associated with young individuals, women, higher education, and having access to the internet at home. However, in multivariate analysis by logistic regression, including variables with p ≤ 0.05, only age remained significant (p < 0.01).

**Table 2:** Demographic characteristics and association with obtaining skin health information in the internet.

Variable	Obtained information, n (%)	Did not obtain information, n (%)	PR (95% IC)	p
Age (mean and SD)	40.2 (13.5)	52.5 (15.6)		**< 0.01**
Sex				**< 0.01**
Female	80(78.4)	25 (54.4)	Reference	
Male	22 (21.6)	21 (45.6)	0.47 (0.29 a 0.77)	
Level of schooling				**< 0.01**
Incomplete Elementary School	13 (12.8)	15 (32.6)	Reference	
Complete Elementary School	12 (11.8)	8 (17.4)	1.38 (0.69 a 2.76)	
Complete or incomplete High School	40 (39.2)	16 (34.8)	1.46 (1.01 a 2.13)	
Complete or incomplete Higher Education	37 (36.6)	6 (13.4)	2.59 (1.29 a 5.19)	
Income				0.81
Up to 1,000 reais	35 (34.3)	15 (32.6)	Reference	
1,000 to 3,000 reais	49 (48)	25 (54.4)	0.93 (0.69 a 1.26)	
> 3,000 reais	18 (17.7)	6 (13)	1.19 (0.55 a 2.58)	
Time commuting				0.89
< 20 minutes	26 (25.5)	12 (26.1)	Reference	
20 to 60 minutes	30 (29.4)	14 (30.4)	0.99 (0.65 a 1.53)	
> 60 minutes	46 (45.1)	20 (43.5)	1.02 (0.74 a 1.41)	
Access to the internet	97 (95.1)	34 (73.9)	1.29 (1.08 a 1.54)	**< 0.01**
Possession of a smartphone	96 (94.1)	39 (84.8)	1.11 (0.97 a 1.27)	0.11

The reliability of the information was not associated with age, sex, education, or income; discussing the results with the physician was directly correlated with schooling (p = 0.01 ‒ chi-square trend) and income (p = 0.05 ‒ chi-square trend); eliciting conflict with the medical conduct was not associated with sex, age, schooling or income; the search for alternative treatments was associated with young individuals (36.82 [12.32] 43.33 [14.17] years; p = 0.02 ‒ Student's *t* test) and a higher level of schooling (p < 0.01 ‒ chi-square trend); and the performance of treatments based on virtual searches was associated only with young individuals (35.85 [13.21] × 42.80 [13.72] years; p = 0.02 ‒ Student's *t* test).

The cluster analysis of information types showed two main independent search patterns, focusing on the diagnosis, treatments, and other information. The analysis of the search portal and tools showed a predominant pattern of search engine use (Figure 1, Figure 2).

**Figure 1:**
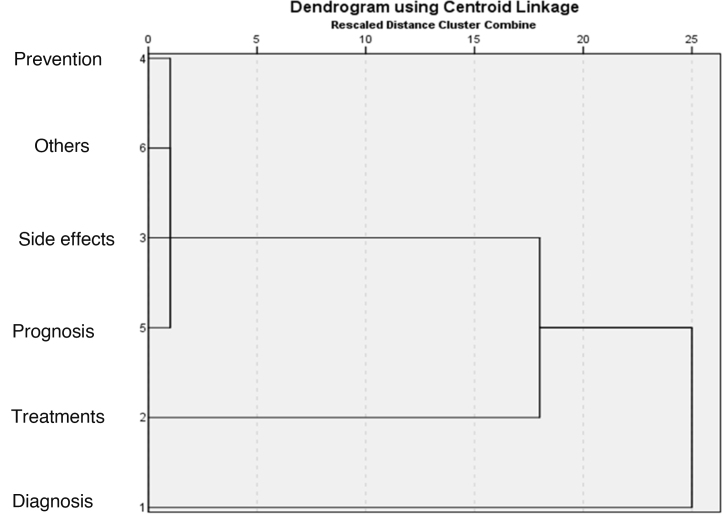
Association patterns between search reasons.

**Figure 2:**
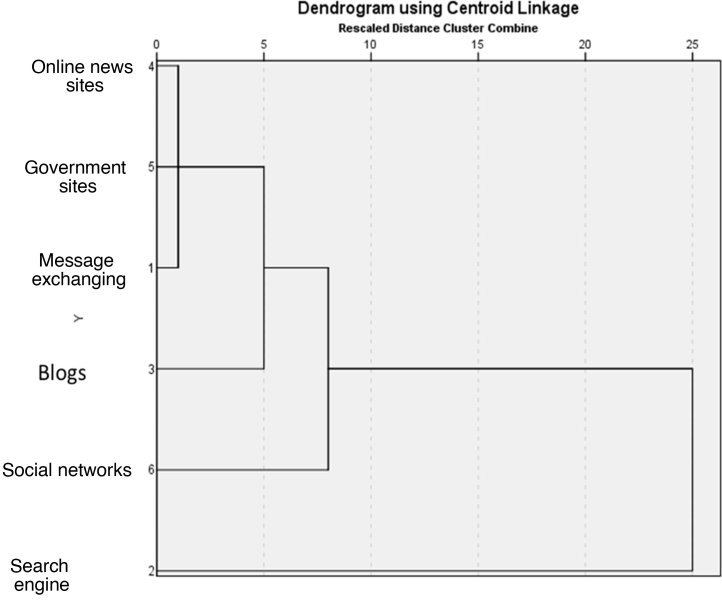
Association patterns between search sources.

It was observed that most of the participants use the internet to carry out health-related searches, corroborating the study hypothesis. A similar article published by the University of Pittsburgh (2015) showed that most participants (74.7%) used the internet to obtain health-related information. However, only a minority used it for dermatological purposes (38.7%).^4^

The present study shows that the search for information took place mainly on smartphones, which may be associated with convenience and easier access.

Although most interviewees use the internet for health-related searches, the obtained information does not have a great deal of influence on most of them. A study conducted in 2015 by the University of Granada described the stability of the bond between doctor and patient, even with the use of virtual tools.^5^

However, it is important to state that approximately 19% of the participants have already undergone some treatment based on virtual searches only, and 2.7% have already abandoned a treatment due to the internet.

As for the reason for the search, the main one was a diagnosis, followed by treatment options, which were sought mainly before the medical appointment. However, 45% of the interviewees sought information even after the appointment, suggesting the persistence of doubts or dissatisfaction with the medical approach.

Although age, sex, schooling, and home access to the internet were associated with the search for information on dermatological health in the bivariate analysis, it should be noted that in the multivariate analysis, only age remained significantly associated with the search for information on skin health. Thus, it is likely that technological, socioeconomic, and cultural factors associated with younger individuals lead them to seek more information about dermatological health on the internet.

It is emphasized that this study was carried out in a center including patients exclusively from the public health system, which may interfere with the validity of the results for the private health system or other regions in the country. However, the results were consistent with other studies on the same topic.

Therefore, it is concluded that the search for information about dermatological health on the internet is frequent, especially among young individuals, albeit with low reliability being attributed to the information. Hence, it is essential to adopt educational measures with the population on where to find reliable information.

## Financial support

None declared.

## Authors' contributions

Bianca Latance da Cruz: Data collection; data interpretation; critical review of important intellectual content; approval of the final version of the manuscript.

Arthur Cesar dos Santos Minato: Data collection; data interpretation; critical review of important intellectual content; approval of the final version of the manuscript.

Ioana Bittencourt Mourão: Data collection; data interpretation; critical review of important intellectual content; approval of the final version of the manuscript.

Dayane Neres Pereira: Data collection; data interpretation; critical review of important intellectual content; approval of the final version of the manuscript.

Miguel Huckembeck de Oliveira: Data collection; data interpretation; critical review of important intellectual content; approval of the final version of the manuscript.

Juliano Vilaverde Schmitt: Design and planning of the study; data collection, or analysis and interpretation of data; critical review of important intellectual content; approval of the final version of the manuscript.

## Conflicts of interest

None declared.

## References

[1] Atkinson N.L., Saperstein S.L., Pleis J. (2009). Using the internet for health-related activities: findings from a national probability sample. J Med Internet Res.

[2] Ybarra M., Suman M. (2008). Reasons, assessments and actions taken: sex and age differences in uses of Internet health information. Health Educ Res.

[3] Moretti F.A., Oliveira V.E., Silva E.M.K. (2012). Acesso a informações de saúde na internet: uma questão de saúde pública?. Rev Assoc Med Bras.

[4] Wolf J.A., Moreau J.F., Patton T.J., Winger D.G., Ferris L.K. (2015). Prevalence and impact of health-related internet and smartphone use among dermatology patients. Cutis.

[5] Orgaz-Molina J., Cotugno M., Girón-Prieto M.S., Arrabal-Polo M.A., Ruiz-Carrascosa J.C., Buendía-Eisman A. (2015). A study of Internet searches for medical information in dermatology patients: The patient-physician relationship. Actas Dermosifiliogr.

